# Quality of Life of Complete Denture Wearers—A Comparative Study between Conventional Dentures and Acrylic Dentures with Vitamin B12 Incorporated

**DOI:** 10.3390/medicina57080820

**Published:** 2021-08-13

**Authors:** Dana Gabriela Budală, Elena Raluca Baciu, Dragoș Ioan Virvescu, Adina Armencia, Mihaela Monica Scutariu, Zinovia Surlari, Carina Balcoș

**Affiliations:** Faculty of Dental Medicine, “Grigore T. Popa” University of Medicine and Pharmacy, 700115 Iași, Romania; dana-gabriela.bosinceanu@umfiasi.ro (D.G.B.); adina.armencia@umfiasi.ro (A.A.); mihaela.scutariu@umfiasi.ro (M.M.S.); zinovia.surlari@umfiasi.ro (Z.S.); carina.balcos@umfiasi.ro (C.B.)

**Keywords:** complete dentures, crosslinked polymerization, vitamin B12, quality of life

## Abstract

*Background and objectives:* This paper is a sequel to the studies that focused on the optimization of the structure of classical acrylates with vitamin B12 as a template and their impact on patients’ general and local health. In this context, we aim to investigate the relationships between attitudes and behavior regarding oral health, oral health status, and quality of life related to oral health in the case of patients with conventional dentures and those with dentures improved with vitamin B12. *Material and Methods:* The sample size was estimated from previous studies, and 252 participants were enrolled and divided into two groups, one of which received as a treatment complete dentures with B12, and the other group was represented by complete edentulous patients treated with conventional complete dentures. The impact of oral health in general on patient satisfaction and quality of life was assessed using Oral Health Impact Profile OHIP-14 questionnaires. The descriptive statistical analysis was performed using SPSS 20.0. A value of *p* < 0.05 was considered statistically significant. *Results:* Differences registered between the two groups were statistically significant in all evaluated dimensions. The largest differences were recorded for physical, psychological, and social disability as well as for the level of disability, all in favor of denture B12 wearers. *Conclusions:* The materials used in the treatment of complete edentation can be a future research direction that can not only improve their mechanical strength but can contribute to maintaining the superior health of the oral mucosa and can also be a way of transporting substances necessary for the normal development of the metabolism of the whole body, such as vitamin B12 in our case.

## 1. Introduction

Complete edentation has concerned medical science since ancient times, and even with progress, both scientific and technological, the rehabilitation of the jaws leaves a degree of functional deficit [1].The establishment and realization of an appropriate treatment of the edentulous state must take into account the structure and functions of the constituent elements of the stomatognathic system and the capacity of and the response to the dental materials, as well as their biological integration [2,3].

The modern trajectories of therapeutic solutions for complete edentation tend towards dentures instead of implants [4], but the clinical reality correlated with the multiple social aspects that remain today and the limits related to a patient’s general condition are two of several arguments that represent important starting points for conducting relevant studies [5,6] to improve the comfort of complete denture wearers by evaluating the used biomaterials, which undergo structural changes [7,8].

This paper is a sequel to the studies on the optimization of the structure of classical acrylic resins and their impact on patients’ general and local health, using classical polymerization derivative techniques, namely, crosslinked polymerization, using vitamin B12 as a template [9]. The great advantage of this method is the formation of memory sites that can be used to transport various medicinal substances.

Vitamin B12 (cyanocobalamin) is a water-soluble vitamin that plays an important role in metabolism in the body, DNA repair, electron transfer, and fatty acid synthesis in cells [10]. An average adult needs about 1–2 μg of vitamin B12 per day, which is not synthesized in the body [11]. Microorganisms are the main source of B12 in nature, and this vitamin exists naturally only in foods of animal origin, where B12 originates from feed or is synthesized by bacteria in the digestive system [12,13]. The prevalence of vitamin B12 deficiency is not known for certain and is very hard to validate because of the diverse etiologies and different assays used to determine serum levels; however, it seems to increase with age. Pennypacker et al. showed the presence of vitamin B12 deficiency (confirmed by laboratory tests in 15% of people over 65 years of age [14]). Other studies have shown that institutionalized elderly people with multiple comorbidities and reduced mobility are more prone to vitamin B12 deficiency (between 30% and 40%) than uninstitutionalized elderly people [15]. Vitamin B12 deficiency can present with several oral manifestations that are considered nonspecific, such as glossitis, glossodynia, recurrent ulcers, lingual paresthesia, burning, pruritus, dysgeusia, intolerance to dental prosthesis, intermittent xerostomia, stomatitis, and cheilitis [16,17].

In this context, the aim of our study was to investigate the quality of life of patients through an oral health impact profile (OHIP-14) in the case of patients with conventional acrylic dentures and those with dentures improved with vitamin B12.

## 2. Materials and Methods

The study group included 252 participants, divided into two equal groups: 126 subjects wearing conventional acrylic dentures and 126 subjects wearing acrylic dentures with vitamin B12 incorporated. The two groups were represented by patients aged between 55 and 85 years, of which 117 were men and 135 women.

The polymerization method used for a half of dentures wearers in this study was a template polymerization. As a template molecule for dentures, we used cyanocobalamin, which is the form of vitamin B12, with widespread use from a clinical point of view, due to its availability and stability. Template polymerization was designed in order to op-timize mass polymerization, to obtain polymers with (highly) crosslinked structure having the characteristics of thermally, mechanically, and chemically resistant materials The process of retention–release of vitamin B12 is a physical process that has been highlighted by obtaining ultraviolet-visible absorption spectra with the help of a UV-Vis Spectrophotometer SPECORD 200, Analytik Jena GmbH Germany. After complete removal of the template, smart polymers have an increased affinity for substances that have a template-like structure, and its retention can be made in much larger quantities than that used in synthesis [9].

In order to determine the patients’ satisfaction with their quality of life, one month after the treatment [18], a subjective evaluation was performed, based on tools for collecting questionnaire data. The Grigore T. Popa University of Medicine and Pharmacy’s Ethics Commission of Scientific Research accepted the study protocol, which was given the number 91/14.06.2021.

The design of the Oral Health Impact Profile (OHIP) tool began from Locker’s theoretical model of oral health, which considered seven dimensions/areas of the impact of oral health on quality of life. Shorter variants with 14 and 20 questions (OHIP-14, OHIP-20), with a smaller number of questions compared to the original version, allow the collection of information in a shorter time without reducing the accuracy of the assessment compared to the original variant [11].

The impact of oral health in general on patient satisfaction and quality of life was assessed using the Romanian version of the OHIP-14 questionnaire [19]. The OHIP-14 contained 14 items in seven dimensions, covering the following factors: functional limitation, physical pain, psychological discomfort, physical inability, psychological inability, social inability, and incapacity [20,21].

The questions contained in all OHIP questionnaires are formulated to measure the effects mentioned in their negative rather than positive dimension. The answers are recorded on a 5-level Likert scale, with the authors indicating a coding of them from 0 to 4 (4—very often, 3—quite often, 2—occasionally, 1—almost never and 0—never), and the answer “I do not know” was coded as a missing value to which a value corresponding to the average of all the values recorded in that question was subsequently assigned. The total score varied depending on the number of questions in the questionnaire. The higher the score, the greater the impact of oral health issues on quality of life.

From each patient’s observation sheets, we extracted information on the occurrence of complications following the wearing of dentures (stomatitis, oral candidiasis) and whether patients followed the dentures hygiene rules, with data recorded by the dentists who performed the prosthetic treatment.

The OHIP-14-dependent variable was dichotomized for the statistical analysis, in which zero values were recorded as the absence of impact and non-zero values as the presence of some impact. The software used for the descriptive statistical analysis of the obtained data was SPSS 20.0 for Windows (SPSS, Inc., Chicago, IL, USA). A Chi square test was used for comparison between groups; *p*-values less than 0.05 were considered statistically significant.

## 3. Results

Tooth loss with the installation of complete edentation leads to a profound impairment of oral status and functional performance—mainly masticatory—but also facial esthetics and has a strong negative impact on the general and nutritional condition and quality of life of the individual concerned.

The analysis of the distribution of subjects by gender showed that the number of female subjects was higher than that of male subjects (135, 53.57%), with 138 of the subjects corresponding to the age category 65–74 years (Table 1).

The evaluation criteria of the study participants included following the hygiene rules of dentures and the presence of mucosal complications in denture wearers. The statistical analysis showed that a relatively small number of participants did not follow the hygiene rules (22.2%), with this being true for more wearers of conventional dentures (15.1%), and the presence of local complications was recorded more often in wearers of conventional dentures (34.1%) (Table 2).

The analysis of the distribution of the answers to the questionnaire of the participants who wore conventional complete dentures showed that more women than men experienced pain more frequently (23%), with this being true for more subjects aged 75–85 years (33.3%) compared with other groups. There were also higher values for the “occasional” and “frequent” response variables for the physical, psychological, and social disability dimensions as well as for the disability dimension (Table 3).

The analysis of the distribution of the answers to the questionnaire for the wearers of dentures with B12 showed that they presented fewer problems, reporting in a very small proportion an unpleasant taste (“occasionally”—1.6% and “frequently”—0.8%) and pain (“occasionally”—15.1% and “frequently”—4%), while for the other dimensions, the highest values were obtained for the answer variant “never” (Table 4). The most affected members of the group were male subjects aged between 55 and 64 years.

Differences registered between the two groups were statistically significant at all evaluated dimensions. The largest differences were recorded for physical pain (difference 0.81, *p* = 0.000), handicap (difference 1.59, *p* = 0.000) and psychological (difference 1.27, *p* = 0.000) and social disability (difference 1.66, *p* = 0.000), as well as for the level of disability, all in favor of wearers of dentures with vitamin B12 (Table 5).

One factor that obviously contributed to the occurrence of various complaints (pain, discomfort) was the lack of proper hygiene regarding the wearing of dentures. In our study, of those who did not follow the rules (washing after each meal and immersion for 1 h in a glass of water with vitamin B12), 57.1% had denture stomatitis and 39.3% suffered candidiasis (Table 6).

## 4. Discussion

Complete dentures, with their shortcomings related to poor balance, functional limitations, reduced masticatory performance, and lesions under dentures, can affect patients’ quality of life.

In general, patients who seek complete denture treatment have lower OHRQoL scores than dentate older people and patients receiving different modalities of prosthodontic treatment [22]. Many studies have shown that providing conventional complete dentures to edentulous patients can improve their appearance, chewing ability, social function, and OHRQoL [22,23,24].

In the literature, there are few studies that describe how to improve acrylic resin by incorporating vitamin B12 [9,25,26]. To the best of our knowledge, this is the first study to examine and compare how a conventional complete denture and a denture with vitamin B12 incorporated might affect a patient’s QoL as measured by the OHIP-14.

Mucosal problems such as stomatitis and oral candidiasis are common among complete acrylic denture wearers. This oral inflammatory pathology predominantly includes symptoms such as erythema, discomfort, burning, and the inflammation of the oral mucosa. The etiology of denture stomatitis includes poor oral and denture hygiene, mucosal trauma resulting from incorrect dentures, nutritional deficiency, metabolic diseases or continuous nighttime wearing of dentures [27,28].

In our study, the frequency of complications was higher in wearers of conventional dentures (stomatitis—21.4%, candidiasis—12.7%) compared to those with vitamin B12, especially for the subjects who did not have proper denture hygiene.

As Candida colonization and denture stomatitis are common problems, efforts have been made to minimize the adherence of Candida to denture bases, including modifications of denture base materials and the addition of antimicrobial agents into denture adhesives. Hence, there is rich literature and numerous published studies on the use of denture bases as a transport vehicle for various substances such as Nistatin, Miconazole, and Clorhexidine [29].

However, maintaining excellent oral health in denture wearers is still problematic and is frequently associated with oral candidiasis, so in this context, our research attempted to find an answer to a problem that is an important point of debate for removable prosthetics; namely, the improvement of the quality of life of denture wearers.

Dentures with vitamin B12 incorporated might be beneficial for edentulous patients with glossitis, angular cheilitis, recurrent oral ulcers, oral candidiasis, widespread erythematous mucositis, recurrent aphthous stomatitis, lichen planus and atrophic glossitis, and a pale oral mucosa, which is associated with cobalamin deficiency [30,31,32].

The recommended daily amount of vitamin B12 for adults is 2.4 micrograms. This dose can be given daily via through the denture if it is kept in a glass of water and vitamin B12 for 8 h to collect 2 mcg of vitamin B12 per day. [9] There are no in vivo studies to indicate the amount of vitamin B12 released from the denture during a day. The fact that during the 8 h used to recharge the denture does not exceed the amount of 2 mcg of vitamin B12 makes this method of administration not present risks to the patient’s health by overdose, with the excess of vitamin B12 being eliminated through urine.

Using tools to assess patients’ quality of life will help healthcare professionals to choose between different alternative treatments, to inform patients about the possible effects of different medical procedures, to monitor the progress of the treatments applied from the patient’s point of view, and, finally, to design effective and efficient health care packages [33].

Future directions of study should target a study group whose general health status should be carefully assessed to eliminate any general conditions, because treatment may not be as effective in certain categories of patients with general psychiatric, digestive or bone resorption illnesses that do not provide the stability of dentures and therefore do not ensure wearing them all the time.

Finally, some of the study’s limitations must be considered, such as the short time period in which the evaluation was conducted (one month after the final treatment), being necessary to carry out a long-term revaluation in order to highlight the benefits of the dentures with vitamin B12; in addition, the low number of studies on improved acrylic resins must be considered. The evaluation of patients’ quality of life one month after the end of treatment was chosen out of the desire to observe how patients adapt to the denture in terms of inflammatory phenomena recurrence that frequently occur in these clinical situations, inflammatory phenomena that can be modulated by the presence of substances with a preventive medicinal effect and also to be able to continue this study with observations collected after a long-time evaluation.

## 5. Conclusions

Based on our findings, improving denture material by introducing vitamin B12 in a polymerizing process can enhance the quality of denture wearers’ lives, leading to less pain sensation and discomfort and reduced numbers of cases of denture stomatitis and candidiasis.

The materials used in the treatment of complete edentation can be a future research direction that can not only improve their mechanical strength but can contribute to maintaining the superior health of the oral mucosa and can also be a way of transporting substances necessary for the normal development of the metabolism of the whole body, such as vitamin B12—an element that patients who are usually in their third age are deprived of—in our case.

## Figures and Tables

**Table 1:** Demographic features of the study group.

Study Group	Conventional Complete Dentures		Complete Dentures with Vitamin B12	
	No.	%	No.	%
**G** **ender**				
Female	72	57.1	63	50.0
Male	54	42.9	63	50.0
**A** **ge group**				
55–64	39	31.0	39	31.0
65–74	69	54.8	69	54.8
75–85	18	14.3	18	14.3

**Table 2:** Distribution of participants according to denture hygiene and local complications.

Denture Hygiene and Local Complications	Conventional Complete Dentures		Complete Dentures with Vitamin B12	
	No.	%	No.	%
**Follow denture hygiene rules**				
**Yes**	107	84.9	117	92.9
**No**	19	15.1	9	7.1
**Complication**				
**No complication**	83	65.9	118	93.7
**Denture stomatitis**	27	21.4	7	5.6
**Candidiasis**	16	12.7	1	0.8

**Table 3:** Distribution of the answers to the OHIP questionnaire for conventional denture wearers.

Conventional Dentures	No.	%	Sex		Group Age		
			Female	Male	55–64	65–74	75–85
**Q1 Trouble pronouncing words correctly**							
Never	43	34.1	29.2	40.7	51.3	26.1	27.8
Almost never	68	54.0	56.9	54.50	48.7	58.0	50.0
Occasionally	15	11.9	13.9	9.3	0.0	15.9	22.2
**Q2 Feeling of bad taste**							
Never	2	1.6	0.0	3.7	5.1	0.0	0.0
Almost never	96	76.2	75	77.8	89.7	72.5	61.1
Occasionally	28	22.2	25	18.5	5.1	27.5	38.9
**Q3 Painful sensation (discomfort, pain)**							
Never	17	13.5	12.5	14.8	10.3	14.5	16.7
Almost never	59	46.8	43.1	51.9	61.5	42.0	33.3
Occasionally	21	16.7	18.1	14.8	17.9	15.9	16.7
Quite often	29	23.0	26.4	18.5	10.3	27.5	33.3
**Q4 Uncomfortable when eating**							
Never	2	1.6	0.0	3.7	5.1	0.0	0.0
Almost never	90	71.4	73.6	68.5	69.2	72.5	72.2
Occasionally	34	27.0	26.4	27.8	25.6	27.5	27.8
**Q5 Awareness or concern for problems with your mouth**							
Never	20	15.9	13.9	18.5	15.4	15.9	16.7
Almost never	70	55.6	56.9	53.7	56.4	55.1	55.6
Occasionally	36	28.6	29.2	27.8	28.2	29.0	27.8
**Q6 Tension, anxiety due to problems with your mouth**							
Never	18	14.3	12.5	16.7	20.5	11.6	11.1
Almost never	74	58.7	61.1	55.6	53.8	60.9	61.1
Occasionally	34	27.0	26.4	27.8	25.6	27.5	27.8
**Q7 Unsatisfactory daily eating (diet) due to problems with your mouth**							
Never	2	1.6	0.0	3.7	5.1	0.0	0.0
Almost never	89	70.6	72.2	68.5	66.7	72.5	72.2
Occasionally	35	27.8	27.8	27.8	28.2	27.5	27.8
**Q8 Interrupting meals due to problems with your mouth**							
Never	19	15.1	13.9	16.7	17.9	13.0	16.7
Almost never	37	29.4	30.6	27.8	33.3	27.5	27.8
Occasionally	70	55.6	55.6	55.6	48.7	59.4	55.6
**Q9 Nervousness or difficulty relaxing due to problems with your mouth**							
Never	19	15.1	13.9	16.7	17.9	13.0	16.7
Almost never	37	29.4	30.6	27.8	33.3	27.5	27.8
Occasionally	70	55.6	55.6	55.6	48.7	59.4	55.6
**Q10 Unsatisfied, embarrassed because of how your mouth looks**							
Never	18	14.3	13.9	14.8	12.8	14.5	16.7
Almost never	58	46.0	47.2	44.4	46.2	46.4	44.4
Occasionally	50	39.7	38.9	40.7	41.0	39.1	38.9
**Q11 Sensitive, irritable due to problems with your mouth**							
Never	18	14.3	12.5	16.7	15.4	13.0	16.7
Almost never	24	19.0	22.2	14.8	20.5	18.8	16.7
Occasionally	50	39.7	38.9	40.7	38.5	40.6	38.9
Quite often	34	27.0	26.4	27.8	25.6	27.5	27.8
**Q12 Difficulty doing your usual work due to problems with your mouth**							
Never	17	13.5	13.9	13.0	12.8	13.0	16.7
Almost never	23	18.3	18.1	18.5	23.1	15.9	16.7
Occasionally	86	68.3	68.1	68.5	64.1	71.0	66.7
**Q13 Feeling of having a less satisfactory life due to problems with your mouth**							
Never	17	13.5	12.5	14.8	10.3	14.5	16.7
Almost never	23	18.3	18.1	18.5	23.1	15.9	16.7
Occasionally	83	65.9	65.3	66.7	66.7	66.7	61.1
Quite often	3	2.4	4.2	0.0	0.0	2.9	5.6
**Q14 Feeling of being unable to lead a normal life due to problems with your mouth**							
Never	16	12.7	12.5	13.0	10.3	13.0	16.7
Almost never	23	18.3	18.1	18.5	23.1	15.9	16.7
Occasionally	83	65.9	63.9	68.5	66.7	66.7	61.1
Quite often	4	3.2	5.6	0.0	0.0	4.3	5.6

**Table 4:** Distribution of the answers to the OHIP questionnaire for the wearers of dentures with B12.

Questions	No.	%	Sex		Group Age		
			F	M	55–64	65–74	75–85
**Q1 Trouble pronouncing words correctly**							
Never	121	96.0	95.2	96.8	97.4	97.1	88.9
Almost never	5	4.0	4.8	3.2	2.6	2.9	11.1
**Q2 Feeling of bad taste**							
Never	36	28.6	28.6	28.6	28.2	29.0	27.8
Almost never	87	69.0	68.3	69.8	69.2	69.6	66.7
Occasionally	2	1.6	3.2	0.0	0.0	1.4	5.6
Quite often	1	0.8	0.0	1.6	2.6	0.0	0.0
**Q3 Painful sensation (discomfort, pain)**							
Never	69	54.8	55.6	54.0	51.3	56.5	55.6
Almost never	33	26.2	27.0	25.4	25.6	27.5	22.2
Occasionally	19	15.1	14.3	15.9	17.9	13.0	16.7
Quite often	5	4.0	3.2	4.8	5.1	2.9	5.6
**Q4 Uncomfortable when eating**							
Never	52	41.3	41.3	41.3	43.6	40.6	38.9
Almost never	74	58.7	58.7	58.7	56.4	59.4	61.1
**Q5 Awareness or concern for problems with your mouth**							
Never	86	68.3	66.7	69.8	66.7	71.0	61.1
Almost never	40	31.7	33.3	30.2	33.3	29.0	38.9
**Q6 Tension, anxiety due to problems with your mouth**							
Never	53	42.1	42.9	41.3	43.6	40.6	44.4
Almost never	73	57.9	57.1	58.7	56.4	59.4	55.6
**Q7 Unsatisfactory daily eating (diet) due to problems with your mouth**							
Never	42	33.3	31.7	34.9	17.9	39.1	44.4
Almost never	74	58.7	60.3	57.1	66.7	55.1	55.6
Occasionally	10	7.9	7.9	7.9	15.4	5.8	0.0
**Q8 Interrupting meals due to problems with your mouth**							
Never	96	76.2	76.2	76.2	74.4	76.8	77.8
Almost never	30	23.8	23.8	23.8	25.6	23.2	22.2
**Q9 Nervousness or difficulty relaxing due to problems with your mouth**							
Never	109	86.5	87.3	85.7	79.5	89.9	88.9
Almost never	17	13.5	12.7	14.3	20.5	10.1	11.1
**Q10 Unsatisfied, embarrassed because of how your mouth looks**							
Never	103	81.7	82.5	81.0	79.5	82.6	83.3
Almost never	23	18.3	17.5	19.0	20.5	17.4	16.7
**Q11 Sensitive, irritable due to problems with your mouth**							
Never	109	86.5	87.3	85.7	79.5	89.9	88.9
Almost never	17	13.5	12.7	14.3	20.5	10.1	11.1
**Q12 Difficulty doing your usual work due to problems with your mouth**							
Never	102	81.0	81.0	81.0	76.9	84.1	77.8
Almost never	24	19.0	19.0	19.0	23.1	15.9	22.2
**Q13 Feeling of having a less satisfactory life due to problems with your mouth**							
Never	125	99.2	98.4	100.0	100.0	100.0	94.4
Almost never	1	0.8	1.6	0.0	0.0	0.0	5.6
**Q14 Feeling of being unable to lead a normal life due to problems with your mouth**							
Never	125	99.2	98.4	100.0	100.0	100.0	94.4
Almost never	1	0.8	1.6	0.0	0.0	0.0	5.6

**Table 5:** The differences registered between the two groups for each dimension evaluated.

Dimension	Means Comparing	Mean Value	SD	*Dif.*	*p*
**Functional limitation**	**Q1 Trouble pronouncing words correctly**				
	Conventional dentures	0.78	0.644	0.74	0.000
	Dentures with vitamin B12	0.04	0.196		
	**Q2 Feeling of bad taste**				
	Conventional dentures	1.21	0.444	0.46	0.000
	Dentures with vitamin B12	0.75	0.521		
**Physical pain**	**Q3 Painful sensation (discomfort, pain)**				
	Conventional dentures	1.49	0.994	0.81	0.000
	Dentures with vitamin B12	0.68	0.873		
	**Q4 Uncomfortable when eating**				
	Conventional dentures	1.25	0.472	0.66	0.000
	Dentures with vitamin B12	0.59	0.494		
**Psychological discomfort**	**Q5 Awareness or concern for problems with your mouth**				
	Conventional dentures	1.13	0.657	0.81	0.000
	Dentures with vitamin B12	0.32	0.467		
	**Q6 Tension, anxiety due to problems with your mouth**				
	Conventional dentures	1.13	0.632	0.55	0.000
	Dentures with vitamin B12	0.58	0.496		
**Physical disability**	**Q7 Unsatisfactory daily eating (diet) due to problems with your mouth**				
	Conventional dentures	1.26	0.476	0.51	0.000
	Dentures with vitamin B12	0.75	0.592		
	**Q8 Interrupting meals due to problems with your mouth**				
	Conventional dentures	1.40	0.739	1.16	0.000
	Dentures with vitamin B12	0.24	0.428		
**Psychological disability**	**Q9 Nervousness or difficulty relaxing due to problems with your mouth**				
	Conventional dentures	1.40	0.739	1.27	0.000
	Dentures with vitamin B12	0.13	0.343		
	**Q10 Unsatisfied, embarrassed because of how your mouth looks**				
	Conventional dentures	1.25	0.692	1.07	0.000
	Dentures with vitamin B12	0.18	0.388		
**Social disability**	**Q11 Sensitive, irritable due to problems with your mouth**				
	Conventional dentures	1.79	0.999	1.66	0.000
	Dentures with vitamin B12	0.13	0.343		
	**Q12 Difficulty doing your usual work due to problems with your mouth**				
	Conventional dentures	1.55	0.722	1.36	0.000
	Dentures with vitamin B12	0.19	0.394		
**Handicap**	**Q13 Feeling of having a less satisfactory life due to problems with your mouth**				
	Conventional dentures	1.57	0.753	1.47	0.000
	Dentures with vitamin B12	0.01	0.089		
	**Q14 Feeling of being unable to lead a normal life due to problems with your mouth**				
	Conventional dentures	1.60	0.750	1.59	0.000
	Dentures with vitamin B12	0.01	0.089		

**Table 6:** Complications crosstabulation hygiene rules.

Complications	Rules of Denture Hygiene		Total
	Following the Rules	Breaking the Rules	
No complication	200	1	201
	89.3%	3.6%	79.8%
Denture stomatitis	18	16	34
	8.0%	57.1%	13.5%
Candidiasis	6	11	17
	2.7%	39.3%	6.7%
**Total**	224	28	252
	100.0%	100.0%	100.0%

## Data Availability

The data that support the findings of this study are available on request from the corresponding author.
